# The association between attitude, self-efficacy, and social support and adherence to diabetes self-care behavior

**DOI:** 10.1186/s13098-018-0386-6

**Published:** 2018-11-27

**Authors:** Mahmood Karimy, Hamid Reza Koohestani, Marzieh Araban

**Affiliations:** 1 Social Determinants of Health Research Center, Saveh University of Medical Sciences, Saveh, Iran; 2 Social Determinants of Health Research Center, Public Health School, Ahvaz Jundishapur University of Medical Sciences, 61375-15751 Ahvaz, Iran; 3 Department of Health Education and Promotion, Public Health School, Ahvaz Jundishapur University of Medical Sciences, Ahvaz, Iran

**Keywords:** Self-care, Self-efficacy, Attitude towards self-care, Social support, Diabetes

## Abstract

**Objectives:**

Diabetes is a chronic illness which requires lifelong self-care behaviors. The objective of the present research was to investigate the association of self-efficacy, attitude and social support with adherence to diabetes self-care behavior.

**Materials and methods:**

In this cross-sectional study conducted in 2017, 403 diabetic patients of Zarandieh, Iran participated. They were evaluated by valid and reliable questionnaires comprised of items on diabetes self-care, self-efficacy in dealing with problems, social support and attitude towards self-care. Data were analyzed using SPSS 18 applying t test, ANOVA, and multiple regression analysis.

**Results:**

The results indicated that patients with higher self-care scores had better self-efficacy, social support, and attitude towards self-care. Moreover, self-efficacy, social support, and attitude towards self-care variables accounted for 39.5% of the total variance of self-care behavior. Furthermore, social support (β = 0.87), self-efficacy (β = 0.52), and attitude towards self-care (β = 0.42) were respectively the most important predictors of self-care behaviors.

**Conclusion:**

Social support, self-efficacy and attitude towards self-care behaviors were associated with self-care behaviors in patient with diabetes. They might suggest that educational programs specifically target these factors.

**Electronic supplementary material:**

The online version of this article (10.1186/s13098-018-0386-6) contains supplementary material, which is available to authorized users.

## Introduction

Diabetes has, more often than not, been a major threat to human health [1]. Based on the reports of world health organization (WHO), the number of patients with diabetes was 425 million in the year 2017 [2], a number which will soar up to 642 million by the year 2040 [3, 4]. Based on a report, the prevalence of this condition among men and women has been estimated to be 9.8% and 11.1%, respectively [5].

There is a close relationship between diabetes as a health risk factor [6] and the modern lifestyle of people [7, 8]. This disease is among the main causes of mortality, disability, and development of other such chronic diseases as renal failure and heart attack [4]. Successful management of diabetes is highly dependent on self-care behaviors of the patient [5, 9] and reducing costs [8, 10, 11]. Most studies have indicated that patients with diabetes have lower self-care ability and suffer from depression, blindness, and foot ulcers [12]. A poor control of diabetes augments long term blood glucose levels which are directly connected with chronic complications such as retinopathy, nephropathy, and certain cardiovascular diseases; such complications require time and high medical expenses in order to be cured [13]. A proper adherence to self-care behaviors can reduce disease complications up to 50% [5]. Non-adherence to self-care practices, on the other hand, is the main cause of mortality in diabetics [14, 15].

Previous studies have reported that that psychological factors like self-efficacy, social support, and attitude influence the behaviors and lifestyles of people [16, 17]. In Bandura’s self-efficacy theory, people who have strong beliefs concerning their abilities are more consistent in keeping up with their daily tasks [16, 18]. Karimy et al. indicated that self-efficacy is the most important predictor of self-care behaviors in patients with diabetes [4]. Social support, as a psychological factor, facilitates healthy behaviors [15]. It has been indicated that although self-care is an individual factor, it is under the influence of social support [19] and has a direct connection with diabetic self-care behaviors [20]. Furthermore, social support improves the ability to adhere to a healthy lifestyle, making individuals more compatible with the disease [19]. Pereira et al. indicated that social support plays the most significant role in blood glucose control in patients with diabetes [21], an issue on which a general agreement is yet to be reached. A study by Storm and Egede indicated that the role of social support components in managing diabetes is still unknown [22]. Studies have also shown that there exists a significant connection between attitude and health behaviors; a positive attitude towards a certain behavior results in more willingness to do that behavior [4, 8, 13]. Therefore, the current study aimed at assessing the association between self-efficacy, attitude towards self-care, and social support and adherence to diabetes self-care behavior in patients with diabetes; also the total variance of adherence to self-care behaviors as explained by these constructs was evaluated.

## Method

### Design, procedure and the study sample

This was a cross-sectional study carried out in from May to September 2017. The population of this study consisted of all 420 patients with diabetes covered by diabetic clinic of Zarandieh, Iran. The inclusion criteria were as follows: age 18 years or more, a prior diagnosis of diabetes by an internal medicine physician, ability to communicate in Farsi (the Iranian official language), and having consented to participate in the study. Seventeen patients were excluded from the study because they failed at either completing the informed consent or filling out the questionnaires. Finally, 403 patients (101 males, and 302 females) were investigated. The body mass index (BMI) was calculated as weight (kg) divided by the squared height (m^2^). The BMI over 30 was considered as obese, between 25 and 29 was defined as overweight, less than 18.5 kg/m^2^ was considered underweight and the BMI between 18.5 and 24.9 was considered normal weight [14, 20].

### Measures

The survey included the following sections and instruments (Additional file 1).

1. *Demographic information sheet*, which includes the information required for age, gender, education level, marital status, employment status, height, weight, duration of the disease, and the sources of information regarding the disease.
2. *Self*-*care questionnaire* the self-care information was gathered using Toobert and Glasgow scale [23]. It is to be noted that this questionnaire have been utilized in several studies in Iran, where its reliability and validity have been approved [4, 24]. In the Toobert and Glasgow self-care questionnaires, the patients report their self-care behaviors in 7 days. It is comprised of 11 questions: “Over the past week, how many days have you taken the recommended diet by physicians?”, “Over the past week, how many days have you monitored your blood sugar?” are among the questions. Higher scores indicate better conditions. The questions are related to blood glucose test (2 questions), sport self-care (2 questions), feet self-care (2 questions), diet self-care (4 questions), and smoking self-care (1 question). Each behavior has a score varying from 0 to 7, with the ultimate score ranging from 0 to 77. The reliability of the scale was confirmed via Cronbach’s alpha in a pilot study, where the amount was found to be satisfactory (0.91).
3. *Self*-*efficacy questionnaire for dealing with problems* This questionnaire consists of 26 questions, such as “When nothing goes well, how much do you think you are able to find the best solution for your problems”; the answer to each item is on an 11 point Likert type scale from never = 0 to I can = 10. This questionnaire has been employed by Mahmoudi et al. in Iran, where its reliability and validity were both corroborated [16]. In our study, the internal consistency of the questionnaire was confirmed, with Cronbach’s alpha equal to 0.89.
4. *Multi*-*dimensional social support questionnaire* This questionnaire consists of 12 questions about friends, family, and important people’s support: “I have friends whom I can share my happiness and sadness with” is among the questions. The answers are on a 7 point Likert type scale from 0 to 6 (completely disagree to completely agree). The validity and reliability of the Persian version have been confirmed in Bagherian-Sararoudi et al. study (Iran) [25]. The reliability of the questionnaire in the current study was confirmed through Cronbach’s Alpha (0.87).
5. *Attitude towards self*-*care* This questionnaire includes 14 items such as, “Daily wash and care of feet is necessary for my diabetes feet ulcer”. The answer to each item is on a 5-point Likert type scale from 1 to 5 (completely disagree to completely agree). The reliability of this questionnaire has been confirmed by Borhani Dizaji et al. [26]. The reliability of the questionnaire in the current study was confirmed as measured by Cronbach’s Alpha (0.83).

### Statistical analysis

All data analyses were conducted according to a pre-established analysis plan through SPSS 18 (SPSS, Inc., Chicago, IL, USA). The data analysis was performed using 18 independent T tests, ANOVA, and multiple regression analysis. To test for the normality of data Kolmogrov–Smirnov test was used; the distributions of the variables approach an underlying normal distribution. An average score was calculated from each scale. Accordingly, so as to predict the outcome variable (diabetes self-care behavior) in this study, the scores of self-efficacy in dealing with problems, social support and attitude towards self-care were included in the regression model.

### Ethical considerations

In order to follow the ethical considerations, all participants were asked to give informed consent. The present study was further approved by the ethics committee of Saveh medical school (Number: IR.SAVEHUMS.1396.17).

## Results

Among the total 403 patients under study, 101 patients (25%) were males with an average age of 58.5 ± 6.2, and 302 patients (75%) were females with an average age of 55.6 ± 3.4. Of all the patients, 91% were married. The average disease duration was 8.9 ± 4 years and 51% of patients were overweight, 21% were obese, and others had a normal body mass index, based on the calculated BMI z-score.

The main sources of information concerning a disease are health care staff (82%), radio and television (67%), friends (59%), internet (49%), and books and magazines (42%).

Table 1 shows the differences in self-efficacy, social support, and attitude towards self-care between/among different groups of demographic variables. Marital status had a significant relationship with self-care behaviors. Regarding the relation between demographic variables with other variables (self-efficacy, social support, and attitude), the results indicated that employment status, gender, duration of the disease, and marital status has a significant relationship with social support, and education level has a significant relationship with attitude (P < 0.05) (Table 1).

**Table 1:** Univariate associations between demographic factors and self-efficacy, social support, and attitude towards self-care scores

Characteristic	N (%)	Attitude towards self-care		Social support		Self-efficacy		Self-care	
		M ± SD	P value	M ± SD	P value	M ± SD	P value	M ± SD	P value
Age	M ± SD 57.5 ± 4.8	30.6 ± 1.7	–	135.5 ± 14.7	–	38.7 ± 5.8	–	38.1 ± 4.7	–
Gender									
Male	101 (25)	29.8 ± 7.8	0.772*	135 ± 33.0	0.21*	35.1 ± 6.8	0.007*	38.2 ± 6.7	0.38*
Female	302 (75)	30.3 ± 9.2		142.7 ± 26.3		39.2 ± 7.4		39.4 ± 6.1	
Marital status									
Married	367 (91)	34.5 ± 8.7	0.01*	141.5 ± 27.8	0.51*	43.1 ± 7.8	0.001*	40.2 ± 5.6	0.46*
Single	36 (9)	28.2 ± 8.3		137.8 ± 26.4		37.2 ± 5.9		39.1 ± 5.3	
Education									
Illiteracy or elementary school	116 (29)	29.2 ± 7.5	0.009**	130.2 ± 26.4	0.001**	34.8 ± 7.6	0.001**	35.6 ± 7.3	0.008**
Secondary	206 (51)	34.8 ± 7.2		137.6 ± 28.3		42.2 ± 5.2		36.2 ± 5.9	
University	81 (20)	35.4 ± 8.5		158.9 ± 27.1		44.6 ± 6.3		41.3 ± 5.1	
BMI									
Normal	113 (28)	31.8 ± 8.9	0.819**	148.8 ± 18.8	0.05**	41.6 ± 7.8	0.71**	40.4 ± 5.8	0.65**
Overweight	205 (51)	30.6 ± 9.1		140.2 ± 19.4		40.3 ± 8.3		38.7 ± 5.5	
Obesity	85 (21)	30.0 ± 8.2		132.8 ± 21.7		39.3 ± 8.7		38.4 ± 7.2	
Occupation									
Housewife	242 (60)	31.0 ± 8.7	0.835**	144.7 ± 31.0	0.810**	40.4 ± 7.6	0.04**	39.2 ± 7.3	0.79**
Employed	69 (17)	31.1 ± 9.2		138.8 ± 28.2		41.7 ± 6.8		40.1 ± 6.0	
Unemployed	12 (3)	29.2 ± 8.6		133.7 ± 23.6		36.9 ± 9.0		38.0 ± 6.8	
Retired	80 (20)	30.5 ± 8.2		137.1 ± 28.0		34.5 ± 8.6		39.2 ± 6.2	
Disease duration (years)									
< 10	301 (75)	31.0 ± 5.9		143.5 ± 27.3	0.47*	41.9 ± 5.8	0.001*	40.3 ± 6.5	0.20*
> 10	102 (25)	30.3 ± 9.3		138.6 ± 27.0		35.8 ± 6.2		38.5 ± 5.1	

*M* mean, *SD* standard deviation

* Using t test

** Using ANOVA

Based on Pearson correlation coefficient test, the three potential predictive factors assessed were all independently and positively associated with self-care behaviors as follows: self-efficacy (P < 0.001, r = 0.39), social support (r = 0.33, P < 0.001), and attitude towards self-care (r = 0.33, P < 0.001). Patients with higher self-care scores had better self-efficacy, social support, and attitude towards self-care.

Multiple regression analysis was used to evaluate the predictive characteristics of self-efficacy, social support, and attitude regarding self-care behaviors of patients with diabetes; results showed that these variables accounted for 39.5% of the variance in self-care behaviors (Table 2).

**Table 2:** Results obtained from multiple linear regression analysis (n = 403)

Model	Beta	P value	95.0% confidence interval for B	
			Lower limit	Upper limit
Social support	0.772	0.001	0.613	1.144
Self-efficacy	0.529	0.001	0.180	0.538
Attitude towards self-care	0.425	0.001	0.143	0.387

Model R^2^ = 0.395

No adjustment was done for demographic variables

## Discussion

At present, considering proper self-care program for patient with diabetes is a key element of care plan [27]. Based on the present results, self-care behaviors were better in married people compared with single ones. Consistent with our results, Didarloo et al. indicated that married people had better self-care behaviors [28]. Also, Dimatteo et al. reported that married patients adhered to healthy diets 1.27 times more than single patients [29]. In the study by Dizaji et al. married people had better self-care behaviors, and more timely attendances to receive services [26]. Such differences between married and single patients are owing to a supportive system such as family and spouse in married patients. Based on our findings, education level had a significant relationship with self-care behaviors, such that with the increase in the level of education, self-care behaviors augmented as well. Consistent with our findings, Glasgow et al. claimed that there existed a significant relationship between self-care and education level in patients with diabetes [30]. Didarloo [28], Ghannadi [31], and Karimi et al. [4] proved that self-care has a significant relationship with education level. Previous findings have indicated that education level is a significant variable in healthy behaviors and efficient management of the disease.

The results of our study indicated that social support is an important and significant predictor for self-care behaviors, consistent with studies done with the aim of evaluating the effects of social support on managing chronic diseases, particularly diabetes [19, 20]. For instance, in Toobert and Glasgow’s study social support was the most important predictor in the treatment adherence of patients with diabetes self-care [23]. Marquez et al. indicated that social support plays an important role in physical activity and weight loss of patients with diabetes [32]. In Shayeghian et al. study social support was associated with a better control of blood glucose and self-care behaviors [33]. Pereira et al. reported that social support had the most important relationship with self-care behaviors such as monitoring blood glucose [21].

Previous studies have shown that self-efficacy is an important precondition for self-management, and plays a critical role in diabetic self-care. The study of Venkataraman in India indicated that self-efficacy is the most important predictor of self-care behaviors in diabetics [34]. In yet another study, self-efficacy had an important role in adhering to self-care behaviors such as physical activity and healthy diets [35]. Contrary to our findings, Hawthorne et al. [36] and Sarkar et al. [37] reported that there exists no significant relationship between self-efficacy and self-management of patients with diabetes. One of the reasons for such inconsistency can be the cultural differences in these two studies (Additional file 1).

Yet another important variable in performing health behaviors is attitude towards self-care. In our study, attitude towards self-care was a significant predictor for self-care behaviors. Didarloo et al. carried out a research on patients with diabetes, which supports our results. They indicated that a positive attitude toward self-care will increase the possibility of better self-care practices [28]. In Pattama et al. study, a significant relationship existed between attitude and self-care behaviors [38]. Ghannadi et al. claimed that positive attitude has a significant relationship with self-care behaviors [31]. According to our results and the results of similar studies [4, 28], it seems that using attitude-improvement strategies might conduce to self-care behaviors.

### Limitations

Due to the nature of the data collection method which is self-report, the data may be subjected to recall bias.

## Conclusion

Social support, self-efficacy, and attitude towards self-care were associated with adherence to self-care behavior among patients with diabetes. Diabetes educators might consider these factors in planning health promotion interventions in order to address the needs of this target group. Due to the cross-sectional nature of the study, causal relationship cannot be determined. Further researches especially randomized controlled trials are needed to confirm the study results.

## Additional file

**Additional file 1.** The survey assessment tool is provided in this additional file.

## Abbreviations

BMI: body mass index
WHO: World Health Organization
