# Supplementary material for: The association between attitude, self-efficacy, and social support and adherence to diabetes self-care behavior
Source: Diabetol Metab Syndr. 2018 Nov 27;10:86. doi: 10.1186/s13098-018-0386-6 (PMC6260748; doi:10.1186/s13098-018-0386-6)
Supplement: Supplementary file 1 — Additional file 1. The survey assessment tool is provided in this additional file. [file 13098_2018_386_MOESM1_ESM.doc]

Research project questionnaire

What is your age? Gender: Male Female

Education level:

Marital status: employment status:

Height: weight: duration of the disease:

Does anyone else in your family have diabetes?

Sources of gathering information about the disease:

**Perceived social support**

Circle the “0” if you Very completely Disagree Circle the “1” if you Strongly Disagree Circle the “2” if you Mildly Disagree Circle the “3” if you are Neutral Circle the “4” if you Mildly Agree Circle the “5” if you Strongly Agree Circle the “6” if you completely Agree

| Significant others | 0 | 1 | 2 | 3 | 4 | 5 | 6 |
| --- | --- | --- | --- | --- | --- | --- | --- |
| There is a special person with whom I can share joys and sorrows |  |  |  |  |  |  |  |
| There is a special person who is around when I am in need |  |  |  |  |  |  |  |
| There is a special person in my life who cares about my feelings |  |  |  |  |  |  |  |
| I have a special person who is the real source of comfort to me |  |  |  |  |  |  |  |
| Family |  |  |  |  |  |  |  |
| I get the emotional help and support I need from my family |  |  |  |  |  |  |  |
| My family is willing to help me make decisions |  |  |  |  |  |  |  |
| My family really tries to help me |  |  |  |  |  |  |  |
| I can talk about my problems with my family |  |  |  |  |  |  |  |
| Friends |  |  |  |  |  |  |  |
| My friends really tries to help me |  |  |  |  |  |  |  |
| I can count on my friends when things go wrong |  |  |  |  |  |  |  |
| I have friends whom I can share my happiness and sadness with |  |  |  |  |  |  |  |
| I can talk about my problems with my friends |  |  |  |  |  |  |  |

When things aren’t going well for you, or when you’re having problems, how confident or certain are you that you can do the following:

**Never = 0**

**Moderately certain can do=5**

**Certain can do=10**

**0 1 2 3 4 5 6 7 8 9 10**

**For each of the following items, write a number from 0 - 10, using the scale above.**

When everything does not go well **for you**, how much do think you **that you can:**

| **Make myself believe that I am capable of solving my problems.** |  |
| --- | --- |
| **I break my problems down into smaller parts.** |  |
| **Do something positive for myself when I feel discouraged**. |  |
| **I sort out what can be changed, and what cannot be changed.** |  |
| **I get emotional support from friends and family.** |  |
| **I find differ­ent solutions for my most challenging problems.** |  |
| **I think about each part of the problem at a time.** |  |
| **I leave my options open when things get stressful.** |  |
| **When confronting a problem, I make an action plan first.** |  |
| **I develop new hobbies or recreations.** |  |
| **Stop myself from being upset by unpleasant emotions** |  |
| **I always try to avoid unpleasant thoughts** |  |
| **I look for something positive for myself in a negative situation.** |  |
| **Keep from feeling sad.** |  |
| **I resist the impulse to act hastily when I am under pressure.** |  |
| **Try to find different solution for my problem if my first solution does not work.** |  |
| Stand my ground and fight for what I want |  |

**When things aren't going well for you, how confident are you that you can:**

| **Make new friends.** |  |
| --- | --- |
| **Leave options open when things get stressful.** |  |
| **Do something positive for myself when I feel discouraged.** |  |
| **I get friends to help me with the things I need.** |  |
| **I try to visualize a pleasant activity or place in unpleasant situation.** |  |
| **I get help from others about what happened.** |  |
| **I Keep myself from feeling lonely.** |  |
| **I try to pray to remain calm when I am under pressure.** |  |
| **I get emotional support from community organizations or re­sources.** |  |

**0 1 2 3 4 5 6 7**

**For each of the following items, write a number from 0 - 7, using the scale above.**

Exercise

On how many of the last SEVEN DAYS did you participate in at least 30 minutes of physical activity? (Total minutes of continuous activity, including walking) 0 1 2 3 4 5 6 7

On how many of the last SEVEN DAYS did you participate in a specific exercise session (such as swimming, walking, biking) other than what you do around the house or as

part of your work? 0 1 2 3 4 5 6 7

Blood Sugar Testing

On how many of the last SEVEN DAYS did you test your blood sugar?

0 1 2 3 4 5 6 7

On how many of the last SEVEN DAYS did you test your blood sugar the number of times recommended by your health care provider?

0 1 2 3 4 5 6 7

Foot Care

On how many of the last SEVEN DAYS did you check your feet? 0 1 2 3 4 5 6 7

On how many of the last SEVEN DAYS did you inspect the inside of your shoes?

0 1 2 3 4 5 6 7

On how many of the last SEVEN DAYS did you dry between your toes after washing?

0 1 2 3 4 5 6 7

Smoking

Have you smoked a cigarette—even one puff—during the past SEVEN DAYS?

0. No

1. Yes.

If yes, how many cigarettes did you smoke on an average day?

Number of cigarette:

Diet

How many of the last SEVEN DAYS have you followed a healthful eating plan?

0 1 2 3 4 5 6 7

On average, over the past month, how many DAYS PER WEEK have you followed your eating plan? 0 1 2 3 4 5 6 7

On how many of the last SEVEN DAYS did you eat five or more servings of fruits and vegetables?

0 1 2 3 4 5 6 7

On how many of the last SEVEN DAYS did you eat high fat foods such as red meat or full-fat dairy products? 0 1 2 3 4 5 6 7

**Attitude toward self-care**

Please indicate that how much you agree/disagree with each of the following sentences.

| Strongly disagree | Disagree | Neither agree/disagree | agree | Strongly agree |  |  |
| --- | --- | --- | --- | --- | --- | --- |
|  |  |  |  |  | Consumption of fruits and vegetables are beneficial in controlling diabetes. | 1 |
|  |  |  |  |  | Quitting smoking is an effective approach to diabetes treatment. | 2 |
|  |  |  |  |  | Lowering the consumption of sweets and fried foods are effective approaches to diabetes control. | 3 |
|  |  |  |  |  | Doing exercise regulary, is an effective approach to diabetes control. | 4 |
|  |  |  |  |  | Daily foot washing is a key element in the prevention of diabetes foot ulcer. | 5 |
|  |  |  |  |  | Stress management is an effective approach to diabetes control. | 6 |
|  |  |  |  |  | Adherence to diabetes insulin injection or taking tablets is effective approaches to diabetes control. | 7 |
|  |  |  |  |  | Weight loss and control help me to better control diabetes | 8 |
|  |  |  |  |  | Regular physician visiting is necessary for controlling my diabetes. | 9 |
|  |  |  |  |  | Monitoring blood glucose levels at home is effective for prevention diabetes complications. | 10 |
|  |  |  |  |  | Monitoring blood pressure at home is effective for prevention diabetes complications. | 11 |
|  |  |  |  |  | wearing shoes at home reduces diabetes ulceration. | 12 |
|  |  |  |  |  | Checking HBA1C could help me better control my diabetes. | 13 |
|  |  |  |  |  | Daily foot care and nail trimming could prevent diabetes foot ulceration. | 14 |
